# The TSN1 Binding Protein RH31 Is a Component of Stress Granules and Participates in Regulation of Salt-Stress Tolerance in *Arabidopsis*

**DOI:** 10.3389/fpls.2021.804356

**Published:** 2021-12-23

**Authors:** Yanan Liu, Shijie Liu, Huiying Shi, Jingyue Ma, Meng Jing, Yuzhen Han

**Affiliations:** ^1^State Key Laboratory of Plant Physiology and Biochemistry, College of Biological Sciences, China Agricultural University, Beijing, China; ^2^Wheat Research Institute, Weifang Academy of Agricultural Sciences, Weifang, China

**Keywords:** TSN1 binding protein, stress granules, DEAD-box RNA helicase, RH31, salt-stress tolerance

## Abstract

Tudor staphylococcal nucleases (TSNs) are evolutionarily conserved RNA binding proteins, which include redundant TSN1 and TSN2 in *Arabidopsis*. It has been showed TSNs are the components of stress granules (SGs) and regulate plant growth under salt stress. In this study, we find a binding protein of TSN1, RH31, which is a DEAD-box RNA helicase (RH). Subcellular localization studies show that RH31 is mainly located in the nucleus, but under salinity, it translocates to the cytoplasm where it accumulates in cytoplasmic granules. After cycloheximide (CHX) treatment which can block the formation of SGs by interfering with mRNP homeostasis, these cytoplasmic granules disappeared. More importantly, RH31 co-localizes with SGs marker protein RBP47. RH31 deletion results in salt-hypersensitive phenotype, while RH31 overexpression causes more resistant to salt stress. In summary, we demonstrate that RH31, the TSN1 binding protein, is a component of plant SGs and participates in regulation of salt-stress tolerance in *Arabidopsis*.

## Introduction

Abiotic stresses affect plant normal growth and lead to crop losses. Plants resist these environmental stresses by regulating mRNA translation and protein synthesis to alter the proteome rapidly in response to various stress signals. Stress granules (SGs) formation is thought to be required for the post-transcriptional regulation of stress-responsive mRNAs (Tsai et al., 2017; Omer et al., 2018).

SGs are condensates of proteins and RNAs assembled *via* liquid–liquid phase separation which involvement plays emerging roles in RNA-related cellular events under various stresses (Ivanov et al., 2019; Lin and Fang, 2021). SGs assembly is initiated by stress-induced eIF2α phosphorylation (Kedersha et al., 1999). When stress is encountered, SGs are formed to save energy by reprograming their translational machinery to allow selective expression of proteins. Some specific mRNAs translation initiation may occur in SGs (Thomas et al., 2011). SGs also function in the mammalian stress response by sequestering mRNAs and allowing for dynamic sorting of mRNAs for translation, storage, or degradation (Vanderweyde et al., 2013). Zebrafish cells lacking SGs will lose the ability to recover once the stress has ended (Zampedri et al., 2016). The components of SGs, AtTZF4, 5 and 6, are involved in light-, abscisic acid-, and gibberellic acid-mediated regulation of seed germination (Bogamuwa and Jang, 2013). SGs component VOZ2 functions as a transcriptional repressor of DREB2A to increase resistance to higher temperatures in *Arabidopsis* (Koguchi et al., 2017). CI and CII small heat shock proteins, which interact with many stress granule proteins, are both important for tolerance to severe heat stress in *Arabidopsis* (McLoughlin et al., 2016). Thus, SGs play critical roles under various stresses.

Tudor staphylococcal nucleases (TSNs) are evolutionarily conserved RNA binding proteins (Gao et al., 2015; Gutierrez-Beltran et al., 2016), which include redundant TSN1 and TSN2 in *Arabidopsis thaliana*. TSN is also a component of SGs (Yan et al., 2014). Previous studies revealed that TSN is essential for stress tolerance and stabilizes stress-responsive mRNAs expession (Dit Frey et al., 2010). Meanwhile, TSN functions in mRNA catabolism and links the formation of SGs and processing bodies (PBs)-cytoplasmic RNA granules which are believed to be sites of mRNA degradation (Arribere et al., 2011; Gutierrez-Beltran et al., 2015). In the latest study, TSN is established with an important role in stress signaling as a docking platform for stress granule proteins (Gutierrez-Beltran et al., 2021). Therefore, TSN is important for plant development and stress tolerance.

In the present study, we identified a binding protein of TSN1, RH31 (Aubourg et al., 1999), which belongs to the DEAD-box RNA helicase (RH) family. RHs function in a variety of RNA metabolism processes by catalyzing unwinding of RNA secondary structure in an ATP-dependent manner and are known as the best candidates for RNA chaperones (Richardson et al., 1998; Tanner and Linder, 2001; Carlotto et al., 2016). Increasing evidence suggests that RHs perform significant functions in innate immunity. As a transcription coactivator, nuclear DExD/H-box helicase 9 plays a critical role in the stimulation of NF-κB-mediated innate immunity against DNA virus infection in mice (Ng et al., 2018). DDX21 translocates from the nucleus to the cytoplasm and participates in the inhibition of Dengue Virus infection (Dong et al., 2016). The DEAD-box RNA helicase 51 aids cell cancer proliferation by regulating cell cycle progression *via* multiple pathways (Wang et al., 2015a). In addition, DEAD-box RHs also play central roles in plant growth, development and stress responses (Liu et al., 2002, 2008; Vashisht and Tuteja, 2006; Kant et al., 2007). In *Sacchromyces cerevisiae*, *translation initiation factor 2* overexpression confers lithium tolerance in galactose medium (Montero-Lomeli et al., 2002). The DEAD-box RH8 positively regulates ABA signaling and increases drought tolerance *via* inhibiting PP2CA activity (Baek et al., 2018). A putative ATP-dependent DEAD-box RH, HVD1, is induced under salt stress, cold stress, and ABA treatment in sorghum (Nakamura et al., 2004).

In this study, we show that RH31 is binding protein of TSN1. We also prove that RH31 is localized to SGs under salt stress. Furthermore, *rh31* mutants display salt-hypersensitive. More importantly, a pathogen-related (PR) gene, *PROAtCAPE3* (Baek et al., 2010), and some salt tolerance-related genes including *ABA-responsive element binding protein 1* (*AREB1*), *Δ1-PYRROLINE-5-CARBOXYLATESYNTHASE 1* (*P5CS1*), and *RD29B* are downregulated in *rh31* mutants. *atcape3* mutants show the similar salt sensitivity to *rh31*. Thus, we conclude that TSN1 binding protein RH31 is a component of SGs and participates in regulation of salt-stress tolerance in *Arabidopsis*.

## Materials and Methods

### Plant Materials and Growth Conditions

*Arabidopsis* ecotype Columbia (Col-0) was used as wild-type controls. Plant growth conditions were same with the conditions described in the previous study (Yan et al., 2014). Col-0 seed batches that were used for phenotype investigation in this work were collected at the same time and stored in the same conditions as the mutant and transgenic seed batches.

### Construction of *rh31* Mutants and Overexpression Lines, Complemented Transgenic Lines, and *atcape3* Mutants

We used CRISPR/Cas9 technology to obtain the knockout mutant. For *rh31*, the lowest homology target (5'-CGAGGAAGAGCTCAGCAATTGGG-3') was selected. For *atcape3*, the lowest homology target (5'-TTGGGGTGGGACCCTTAAGATGG-3') was selected. Above targets were cloned into the pHEE2A-TRI vector (Wang et al., 2015b). Then, the construct was transformed into Col-0. The *RH31* and *PROAtCAPE3* fragments were amplified using the primers listed in Supplementary Table S1, and homozygotes were selected by restriction enzyme digestion and sequencing from the T_1_ generation. A specific pair of primers (zCas9-IDF3-2/rbcS_E9t-IDR) was used to identify non-transgenic lines of T_2_ generation. These non-transgenic lines were further examined on plates containing 25 mg L^−1^ hygromycin.

The full-length *RH31* genomic sequence was amplified using the primers RH31-OE-LP and RH31-OE-RP (Supplementary Table S1) and then cloned into Super Promoter 1300 vector (pSuper 1300) to obtain overexpression lines (*OE*s).

The RH31-GFP construct described below was transformed into *rh31* lines to generate *RH31* complemented lines, and transgenic line (4–2) with similar mRNA levels as Col-0 was used for further study.

### Plasmid Constructs and Subcellular Localization Assay

In subcellular localization assay, the full-length *RH31* genomic sequence without the stop codon was amplified using the primers RH31-GFP-LP and RH31-GFP-RP (Supplementary Table S1), and then cloned into pSuper 1300-GFP vector. The resulting construct RH31-GFP was transformed into Col-0, and homozygotes were selected from the T_3_ generation, then the roots of 7-day-old seedlings were used to test subcellular localization of RH31. To test the effect of cycloheximide (CHX), 7-day-old seedlings were incubated in liquid medium (0.5 × Murashige and Skoog salts, 1% sucrose, and 0.5 g L^−1^ MES, pH 6.8) supplemented with 200 μg ml^−1^ CHX (Sigma-Aldrich) and shaken for 80 min as previously described (Goeres et al., 2007). To further confirm the localization of RH31, the RH31-GFP construct was used for transient expression assay, and GFP fusion proteins were observed with a Zeiss 710 Meta laser scanning confocal microscope (Zeiss, Oberkochen, Germany) as previously described (Kim and Somers, 2010). For CHX experiments, 100 μg ml^−1^ CHX was added to the respective protoplast suspension, and then gently mixed and incubated for 15 min. The cells were then either kept under control condition or subjected to 150 mM NaCl stress (Weber et al., 2008).

### GST Pull-Down Assay

**Figure 1:**
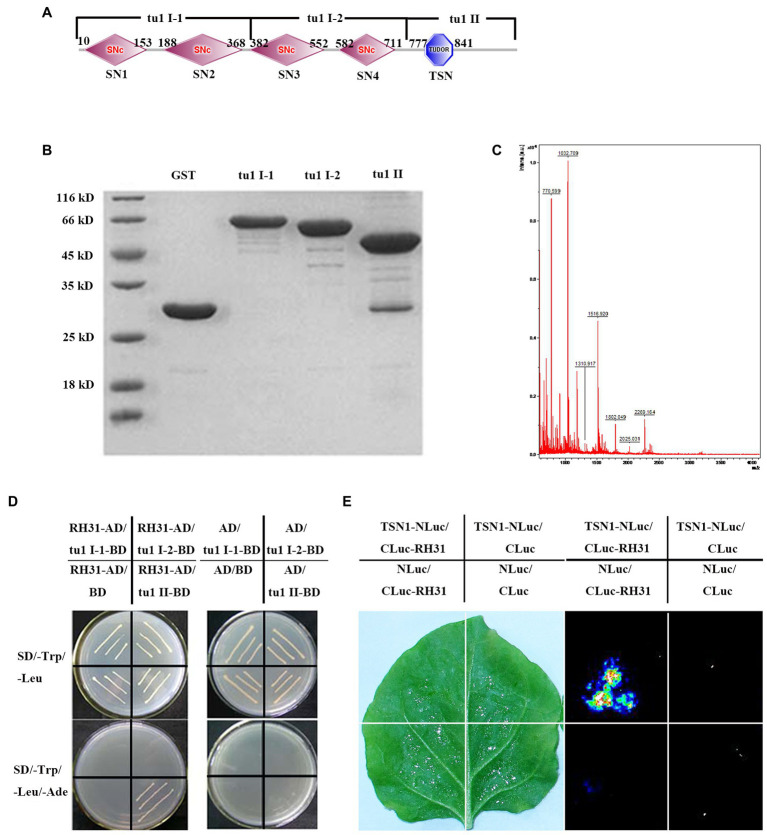
Identification of the RH31-TSN1 interaction. **(A)** The predicted protein domains of TSN1. The four SNc from left to right represent SN1, SN2, SN3, SN4, respectively. **(B)** GST Pull-down assay of truncated TSN1 protein. Three TSN1 fragments were generated as follows: tu1 I-1 (1–380 amino acids), including SN1 and SN2 domains; tu1 I-2 (381–720 amino acids), including SN3 and SN4 domains; tu1 II (721–970 amino acids), including C-terminal TSN (Tudor-SN) domain. GST fusion protein was used as bait to pull down the binding proteins of truncated TSN1 protein among total proteins of *Arabidopsis* seedlings. GST protein was used as a negative control. **(C)** Mass spectrometry analysis of tu1 II binding proteins. **(D)** Binding of tu1 II to RH31 by yeast-two-hybrid analysis. **(E)** Binding of TSN1 to RH31 by firefly luciferase complementation imaging.

Three TSN1 fragments^1^ were generated as follows: tu1 I-1 (1–380 amino acids), including SN1 and SN2 domains and has a molecular weight of approximately 41.4 kD; tu1 I-2 (381–720 amino acids), including SN3 and SN4 domains and has a molecular weight of approximately 37.2 kD; tu1 II (721–970 amino acids), including C-terminal TSN (Tudor-SN) domain and has a molecular weight of 27.5 kD (Figure 1A). These three fragments were amplified using primers listed in Supplementary Table S1, then inserted into pGEX-4 T-1 vector and termed pGEX-tu1 I-1, pGEX-tu1 I-2 and pGEX-tu1 II, respectively. *E. coli* DE3 transformants were induced to express the fusion protein at 26°C for 8 h by the addition of IPTG to 1 mM. The expression of total GST fusion protein was detected using 10% SDS-PAGE. Protein concentration was determined using the Bio-Rad protein assay kit (Bio-Rad, Hercules, CA, United States), using bovine serum albumin as the standard. Buffer L (50 mM Tris–HCl, pH 8.0; 250 mM NaCl; 1 mM EDTA, pH8.0; and pH7.5) balanced Glutathione-Sepharose 4B were incubated with 100 μg GST fusion protein for 1 h at 4°C, and GST protein was used as negative control. Beads were centrifuged at 1000 × g for 2 min at 4°C. After removing the supernatant, beads were washed 3 × 5 min with Buffer L. Total protein of *Arabidopsis thaliana* seedlings was extracted from 0.5 g 2-week-old Col-0 lines using a Plant Protein Extraction Kit (CWBio, Beijing, China) according to manufacturer instructions. After sedimentation at 4°C for 1 h, the solution was centrifuged at 12000 × g for 30 min at 4°C, supernatants were collected, and again centrifuged. Total proteins of *Arabidopsis thaliana* seedlings were added into agarose, then the mixture was incubated overnight at 4°C; the mixture was centrifuged at 1000 g for 2 min at 4°C, after the supernatant was removed, the mixture was washed 4 × 5 min with Buffer L. Agarose was diluted with 100 μl Buffer L, and binding proteins were detected by 10% SDS-PAGE. The gel containing the binding proteins was cut off, and proteins were digested with trypsin and analyzed with LC–MS (Biological Mass Spectrometry Platform of China Agricultural University, Beijing, China). LC–MS data were searched using Matrix Server software against *Arabidospsis* TAIR10_pep_20101214 database.

### Yeast Two-Hybrid Assay

The full-length *RH31* genomic sequence was amplified using the primers RH31-hy-LP and RH31-hy-RP (Supplementary Table S1) and inserted into pGADT-7 vector. The construct was termed as RH31-AD. The tu1 I-1, tu1 I-2, and tu1 II fragments were digested with *Eco*RI and *Bam*HI and then inserted into pGBKT-7 vector. The resulting constructs were termed as tu1 I-1-BD, tu1 I-2-BD, and tu1 II-BD, respectively. Empty pGADT-7 and pGBKT-7 vectors were used as negative controls. Constructs were co-transformed into yeast strain AH109. Yeast cells were cultured at 30°C on SD/−L-T plates. Positive clones were transformed on the selective medium SD/−L-T-A.

### Luciferase Complementation Imaging Assay

The full-length *TSN1* and *RH31* genomic sequences were amplified using the primers listed in Supplementary Table S1, then inserted into NLuc and CLuc vectors, respectively. Empty NLuc and CLuc vectors were used as negative controls. Constructs were co-transformed into *A. tumefaciens* strain GV3101. The Luciferase Complementation Imaging (LCI) assay was performed as previously described (Chen et al., 2008). Bacteria containing CLuc-RH31, TSN1-NLuc, and P19 (Shuhua Yang, State Key Laboratory of Plant Physiology and Biochemistry, China Agricultural University) were centrifuged together and the bacterial mixture was resuspended with activation buffer containing 10 mM MES, 10 mM MgCl_2_, and 150 mM acetosyringone to a final concentration of OD_600_ = 0.5, then incubated at 28°C for 3 h. Bacterial suspensions were infiltrated into young but fully expanded leaves of *N. benthamiana* plants using a needleless syringe. After infiltration, plants were placed at 23°C for 3 days. CCD imaging was used to measure LUC activity.

### Quantitative and Semi-quantitative RT-PCR Analysis

Total RNA was isolated from *Arabidopsis* as previously described (Oñatesánchez and Vicentecarbajosa, 2008). M-MLV reverse transcription system (TaKaRa, Beijing, China) was used to synthesize first-strand cDNA according to the manufacturer’s protocol. Quantitative RT-PCR was conducted using the SYBR Green I Master Mix (TaKaRa, Beijing, China) in a total reaction volume of 20 μl. The reaction was completed on the ABI7500 Fast Real-Time PCR system (Applied Biosystems, Foster City, CA, United States), and data were normalized with respect to *At4g34270* (Czechowski et al., 2005). For semi-quantitative PCR, reactions were performed using Taq DNA polymerase (TaKaRa, Beijing, China) for 30 cycles in a total reaction volume of 25 μl. *Actin2* was used as an internal control. The primers used for quantitative RT-PCR and semi-quantitative PCR are listed in Supplementary Table S1.

### Root Elongation and Fresh Weight Measurement

After 24 h of growth at 22°C on a 16-h light/8 h dark photoperiod on MS agar medium, synchronized growth seedlings (same root length) were transferred to MS medium for another 6 days or MS medium containing 150 mM NaCl or 250 mM mannitol for another 10 days. The root lengths of 7-day-old (normal) or 11-day-old (stress) seedlings were measured. We calculated relative root growth, which was determined as the ratio of root length under stress to root length under normal condition following a previously described method (Ezaki et al., 2007). Experiments were repeated three times with similar results, and at least 30 seedlings per genotype were measured. For fresh weight determination, the weight of at least 20 seedlings was measured. The relative fresh weight under stress conditions was calculated.

### Seedling Survival Assay

Seeds from the Col-0, *rh31* and *OE*s were grown on 1/2 MS medium for 6 days and then transferred onto 1/2 MS medium supplemented with 200 mM NaCl for 5 days (Zhou et al., 2017). Experiments were repeated three times with similar results, and at least 30 seedlings for each genotype were examined per replicate. We calculated seedlings survival ratio according to visibly green leaves (Achard et al., 2006): seedlings with green leaves were counted as living, while seedlings with bleached white or albino leaves were counted as dead. Cell death in leaves was examined by trypan blue staining according to the method described by Bowling et al. (1997).

### Subcellular Localization of RH31-GFP and RBP47-RFP

The RFP-RBP47 vector was generously provided by Dr. Markus Fauth (Weber et al., 2008). *Arabidopsis* mesophyll protoplasts were prepared from rosette leaves of 4-week-old Col-0 plants as previously described (Kim and Somers, 2010). *Arabidopsis* protoplasts were co-transformed with RH31-GFP and RFP-RBP47 and then incubated at 22°C for 20 h. Co-localization analysis was performed using a Zeiss 710 Meta laser scanning confocal microscope (Zeiss, Oberkochen, Germany) as previously described (Kim and Somers, 2010).

## Results

### RH31 Interacts With TSN1 *in vitro* and *in vivo*

Yan et al. (2014) have previously proved that the RNA binding protein TSN1 is a component of stress granules (SGs) and involves in salt-stress adaptation (Yan et al., 2014). Here, TSN1-interacting proteins were identified. Three TSN1 fragments, tu1 I-1 which including SN1 and SN2 domains, tu1 I-2 which including SN3 and SN4 domains, tu1 II which including C-terminal TSN (Tudor-SN) domain were generated (Figure 1A). Several GST tagged TSN1 fragments (pGEX-tu1 I-1, pGEX-tu1 I-2 and pGEX-tu1 II) were extracted, and pull-down assays were performed to obtain the TSN1 binding proteins among total proteins of *Arabidopsis* seedlings (Figures 1A,B). We found that there are some proteins that could bind to pGEX-tu1 II, but no protein could bind to pGEX-tu1 I-1 or pGEX-tu1 I-2 (Figure 1B). A matching protein, which is a DEAD-box RNA helicase (At5g63630), RH31, was showed by mass spectrometry analysis (Figure 1C).

To further confirm the interaction between TSN1 and RH31, yeast two-hybrid assay was performed. When yeast cells were transformed with RH31-AD, tu1 I-1-BD, tu1 I-2-BD or tu1 II-BD, no colonies grew on SD/−L-T-A, while all transformants could grow on SD/−T plates. These results indicate that the transformants cannot self-activated and this yeast two-hybrid system can be used in our subsequent experiment. Then, RH31-AD was co-transformed with tu1 I-1-BD, tu1 I-2-BD, or tu1 II-BD. We found that only bait cells containing both the TSN1 C-terminal fragment tu1 II domain and RH31 could grow on the selective medium SD/−L-T-A, whereas other transformants could not grow (Figure 1D). These results suggest that RH31 can specially interact with C-terminal region of TSN1 *in vitro*.

The LCI assay further verified the interaction between RH31 and TSN1 *in vivo*. We found that co-infiltration of *Agrobacteria* containing CLuc-RH31 and TSN1-NLuc resulted in LUC complementation, while no LUC complementation was produced by expression of TSN1-NLuc construct and the empty 35STCLuc vector, CLuc-RH31 construct and the empty 35STNLuc vector, or empty 35STCLuc vector and empty 35STNLuc vector. These results demonstrate that RH31 can interact with TSN1 both *in vitro* and *in vivo*.

### RH31 Accumulates in SGs Under Salinity

**Figure 2:**
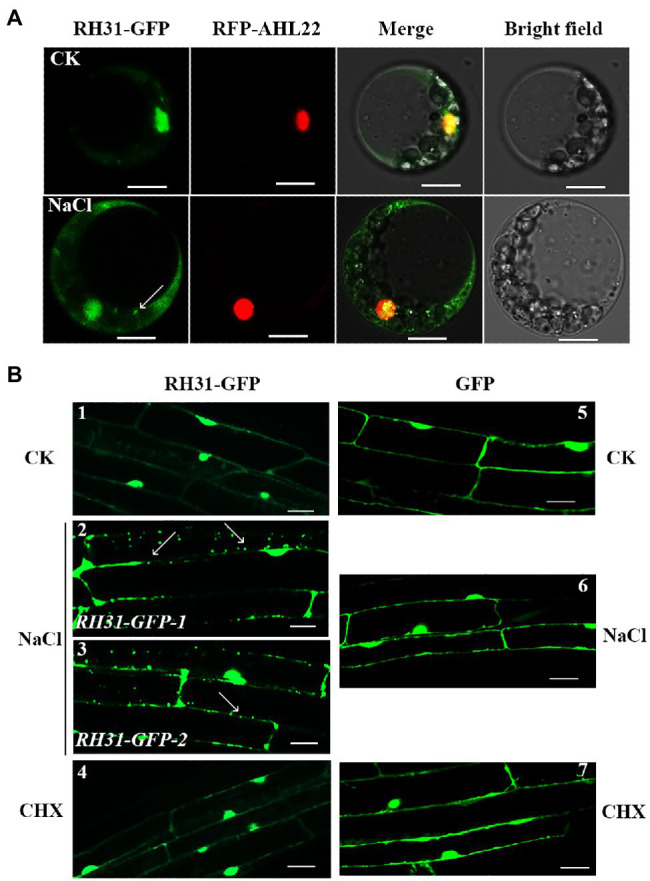
Subcellular localization of RH31-GFP. **(A)** Intracellular distribution of RH31-GFP and RFP-AHL22 in living *Arabidopsis* protoplasts. RH31-GFP mainly distributed in cell nucleus under normal condition (top row), but rapidly shifted to the cytoplasm and redistributed to small granules after 5 min treatment with 150 mM NaCl (bottom row). Scale bars = 10 μm. **(B)** Confocal micrographs of root elongation zones of 7-day-old seedlings. RH31-GFP mainly localized to cell nucleus under normal condition (1), but shifted to the cytoplasm and formed a number of small granules when seedling roots were treated with 150 mM NaCl (2 and 3). *RH31-GFP* seedling roots under salinity no longer showed small granules after treated with CHX (4). *GFP* transgenic lines under normal (5), salt stress (6), and CHX (7) conditions were used as controls. Scale bars = 20 μm. CK, Control; CHX, Cycloheximide. (2) and (3) represent two *RH31-GFP* lines. Arrows point to the granules.

To investigate the subcellular localization of RH31, we constructed a RH31-GFP vector under control of the cauliflower mosaic virus (CaMV) 35S promoter (Weber et al., 2008; Koguchi et al., 2017) and expressed RH31 protein in *Arabidopsis* protoplasts and the *RH31-GFP* transgenic lines, respectively. The transgenic plants rescued the short root length and low fresh weight phenotype of *rh31* mutant which indicated that RH31 protein was expressed (Supplementary Figure S4). RH31-GFP was transiently co-expressed with a nucleus-localized protein, red fluorescent fusion protein RFP-AHL22, in *Arabidopsis* protoplasts (Xiao et al., 2009). Under normal condition, RH31-GFP was mainly distributed in nucleus (Figure 2A, upper). However, after a short term treatment of salt, it rapidly shifted to the cytoplasm and redistributed to small granules (Figure 2A, below). Similarly, RH31-GFP mainly localized in cell nucleus in *RH31-GFP* transgenic lines under normal condition (Figure 2B, sample 1), but it shifted to the cytoplasm and formed some small granules under 150 mM NaCl treatment (Figure 2B, samples 2 and 3). Whereas GFP alone was present throughout the cell under all conditions (Figure 2B, samples 5 and 6).

Previous studies showed that TSN1 accumulates in SGs under stress (Yan et al., 2014). Since RH31 is a binding protein of TSN1, it is possible that the granules we observed were SGs in the *RH31-GFP* transgenic lines. SGs assembly depends on the release of untranslated mRNPs from polysomes. As an inhibitor of the translocation step during the elongation phase in protein synthesis, CHX traps mRNA in polysomes to block mRNA release and inhibits SG assembly in mammalian and plant cells (Kedersha et al., 1999; Weber et al., 2008; Grousl et al., 2009; Gutierrez-Beltran et al., 2015). As we expected, after CHX treatment, there is not the localized spots can be detected in the RH31-GFP transgenic lines under salinity (Figure 2B, sample 4).

**Figure 3:**
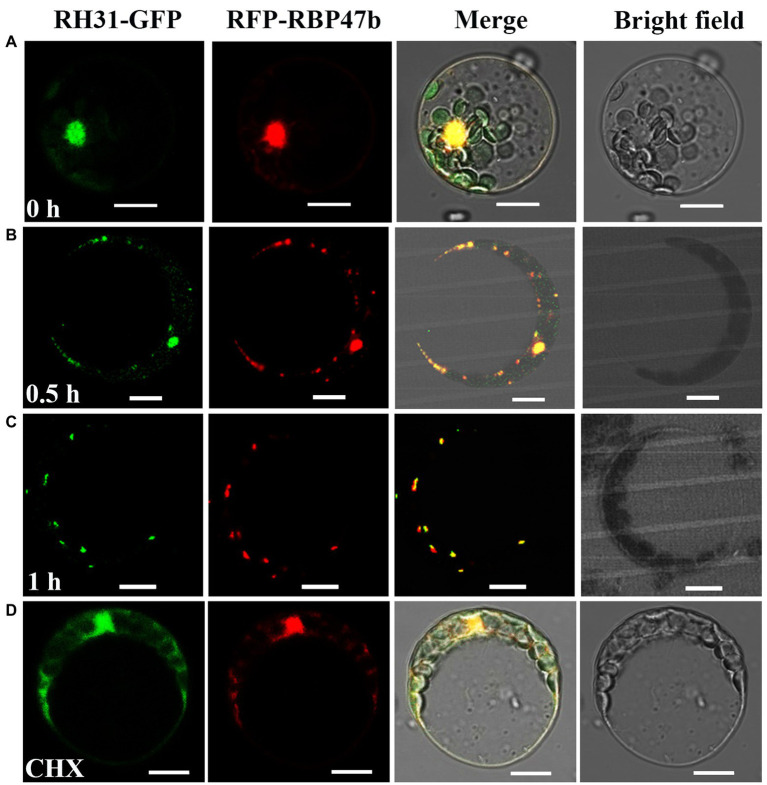
Co-localization analysis of RH31-GFP and RFP-RBP47 in SGs under salt stress in *Arabidopsis* mesophyll protoplasts. **(A)** RH31-GFP and RFP-RBP47 mainly located in nucleus under normal condition. Scale bars = 10 μm. **(B,C)** RH31-GFP and RFP-RBP47 relocated to cytoplasmic granules in response to 150 mM NaCl in different time point, and RH31-GFP fluorescence overlapped with RFP-RBP47 fluorescence. Scale bars = 10 μm. **(D)** After CHX treatment, there were no cytoplasmic granules when treated protoplasts with 150 mM NaCl. Scale bars = 10 μm. CHX, Cycloheximide.

To further confirm that these granules were indeed SGs in *RH31-GFP* transgenic lines, we co-expressed RH31-GFP and RFP-RBP47 under salt stress in *Arabidopsis* mesophyll protoplasts (Figure 3). RFP-RBP47 was primarily located in the nucleus (Figure 3A, lane 2), but was relocated to cytoplasmic granules with 150 mM NaCl treatment (Figures 3B,C, lane 2), which was consistent with previous study (Weber et al., 2008). Similarly, RH31-GFP was also primarily located in the nucleus under normal condition (Figure 3A, lane 1), but shifted into cytoplasmic granules in response to salt stress (Figures 3B,C, lane 1). Moreover, RH31-GFP fluorescence overlapped with RFP-RBP47 fluorescence. After CHX treatment, these cytoplasmic granules disappeared (Figure 3D). These results indicate that RH31 is a component of plant SGs and accumulates in SGs under salt stress.

### Generation of *rh31* Mutant and *RH31* Overexpression Lines

**Figure 4:**
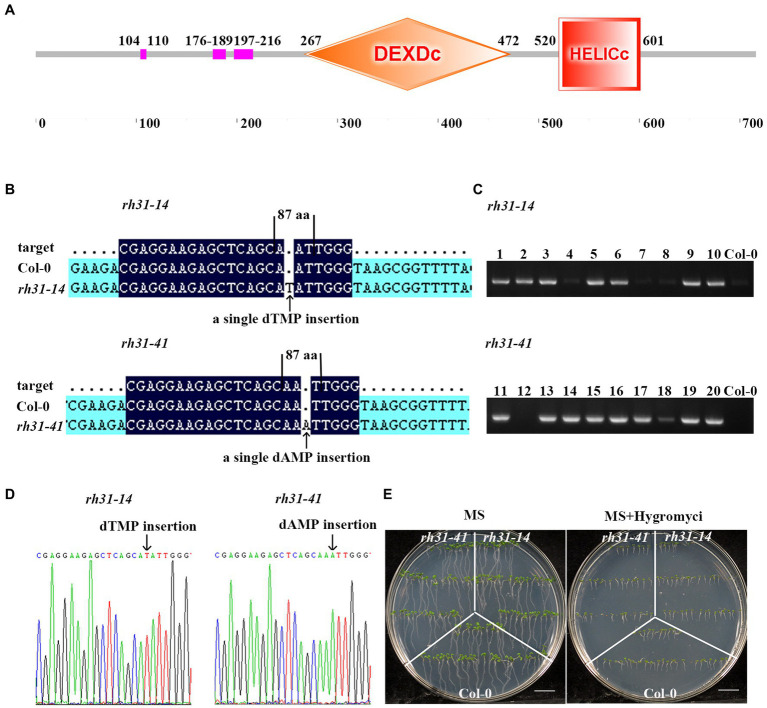
Generation of *rh31* mutants. **(A)** The predicted protein domains of RH31. **(B)** Schematic of the *rh31* insertion mutation. Arrows point to the insertion site. **(C)** RT-PCR analysis of non-transgenic lines. Col-0 genomic DNA was served as a negative control. Plants 4, 7, 8 of *rh31-14* and plant 12 of *rh31-41* were non-transgenic, while other plants were Cas9 transgenic. **(D)** SANGER sequencing chromatography confirming insertion in *rh31* mutants. Arrows point to the insertion site. **(E)** Screening non-Cas9 transgenic plants by hygromycin resistance. The details of target construction and mutant generation are described in the Materials and Methods section. Scale bars = 1 cm.

To determine the role of RH31 under stress, *rh31* mutant and RH31 *OEs* were generated. In terms of *rh31* mutant, the lowest homology sgRNA-targeted *RH31* was cloned into pHEE2A-TRI vector and then was transformed into Col-0. Mutants where loci were located upstream of the functional domain were selected (Figure 4A).^2^ Homozygous *rh31* lines were identified among T_2_ progeny by sequencing. A single dTMP insertion was found within the 87th amino acid codon in *rh31-14* mutant, while a single dAMP insertion was found within the 87th amino acid codon in *rh31-41* mutant (Figures 4B,D); these two mutant loci were both upstream of the functional domain. A specific pair of primers (zCas9-IDF3-2/rbcS_E9t-IDR) was used to exclude any effect of the *Cas9* gene (Figure 4C). We further confirmed hygromycin resistance in these lines (Figure 4E). The selected non-hygromycin resistant lines were used for further study.

In terms of RH31 *OEs*, full length *RH31* gene was introduced into *Arabidopsis* under control of CaMV 35S promoter. Four *RH31-OE*s were confirmed by both RT-PCR and qRT-PCR. *OE1* and *OE2* in which the transcript level of *RH31* was increased by about 10-fold and 76-fold, respectively, were selected for further study (Supplementary Figure S1).

### *RH31* Deletion Results in Increased Sensitivity to Salt, but Not Osmotic Stress, During Early Seedling Growth

**Figure 5:**
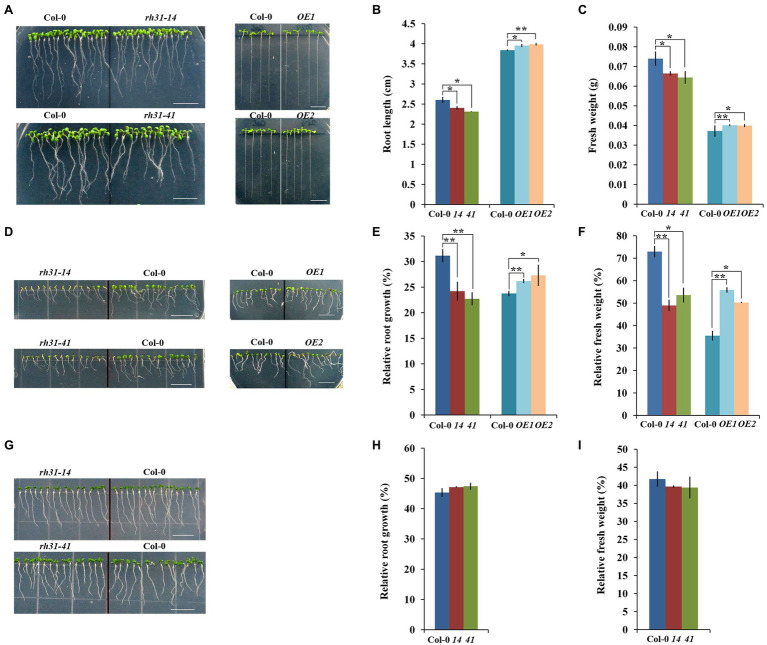
Growth phenotype characterization of *rh31* and *RH31* overexpression (*OE*1, *OE*2) lines under normal and stress conditions. **(A)** Phenotype of 7-day-old *rh31* and 11-day-old *OE* seedlings under normal condition. After germinated for 24 h under normal condition, synchronized growth of Col-0, *rh31*, and *OE* seedlings were transferred to MS medium for another 6 days (*rh31*) or 10 days (*OE*). Experiments were repeated three times with similar results. Scale bars = 1 cm. **(B)** Root length of seedlings shown in **(A)**. The primary roots of at least 30 seedlings were measured and reported as the mean length (*n* = 3 replicates). **(C)** Fresh weight of 11-day-old *rh31* and *OE* seedlings under normal condition. 40 (*rh31*) and 20 (*OE*) seedlings were measured per replicate (*n* = 3 replicates). **(D)** Phenotype of *rh31* and *OE* seedlings treated with 150 mM NaCl for 10 days. After germinated for 24 h under normal condition, synchronized growth of Col-0, *rh31*, and *OE* seedlings were transferred to MS medium with or without 150 mM NaCl for another 10 days. Experiments were repeated three times with similar results. Scale bars = 1 cm. **(E)** Relative root growth of seedlings shown in **(D)**. The primary roots of at least 30 11-day-old seedlings were measured and relative growth was reported as the mean length (*n* = 3 replicates). **(F)** Relative fresh weight of seedlings shown in **(D)**. 40 (*rh31*) and 20 (*OE*) seedlings were measured per replicate, and relative fresh weight was reported as the mean fresh weight (*n* = 3 replicates). **(G)** Phenotype of *rh31* seedlings treated with 250 mM mannitol for 10 days. After germinated for 24 h under normal condition, synchronized growth of Col-0 and *rh31* seedlings were transferred to MS medium with or without 250 mM mannitol for another 10 days. Experiments were repeated three times with similar results. Scale bars = 1 cm. **(H)** Relative root growth of *rh31* seedlings shown in **(G)**. The primary roots of at least 30 11-day-old seedlings were measured and relative growth was reported as the mean length (*n* = 3 replicates). **(I)** Relative fresh weight of seedlings shown in **(G)**. 40 seedlings were measured per replicate, and relative fresh weight was reported as the mean fresh weight (*n* = 3 replicates). ^*^p < 0.05 and ^**^p < 0.01 (Student’s *t*-test) indicate significant differences between *rh31*, *OE*, and Col-0 plants. Error bars indicate the standard error for the average of three independent experiments.

As the above studies showed that RH31 localized to SGs under salt stress, we hypothesized that RH31 might function in stress responses. To test this, synchronized growth of Col-0, *rh31*, and *OE*s were transferred to MS medium with or without NaCl or mannitol treatment. First of all, we designed serial concentrations of NaCl (0, 50, 100, 150, 200, 250 mM) for growth and survival experiments (data not shown), and the most obvious effects of 150 mM and 200 mM concentrations were determined for growth experiment and survival experiment, respectively. Under normal condition, *rh31* seedlings had shorter primary root lengths and reduced fresh weight compared to Col-0, while *OE*s showed increased root growth and biomass accumulation (Figures 5A-C). Moreover, under salt stress (150 mM NaCl), the *rh31* lines were even more severely impaired with distinct small, yellowish cotyledons, obviously shorter primary roots and less fresh weight after 10 days of NaCl treatment, while *OE*s were more resistant to NaCl (Figures 5D–F; Supplementary Figure S2). However, under 250 mM mannitol treatment, no significant difference was observed (Figures 5G–I). These show that *rh31* mutant is relatively sensitive to salt, but not sensitive to osmotic stress.

**Figure 6:**
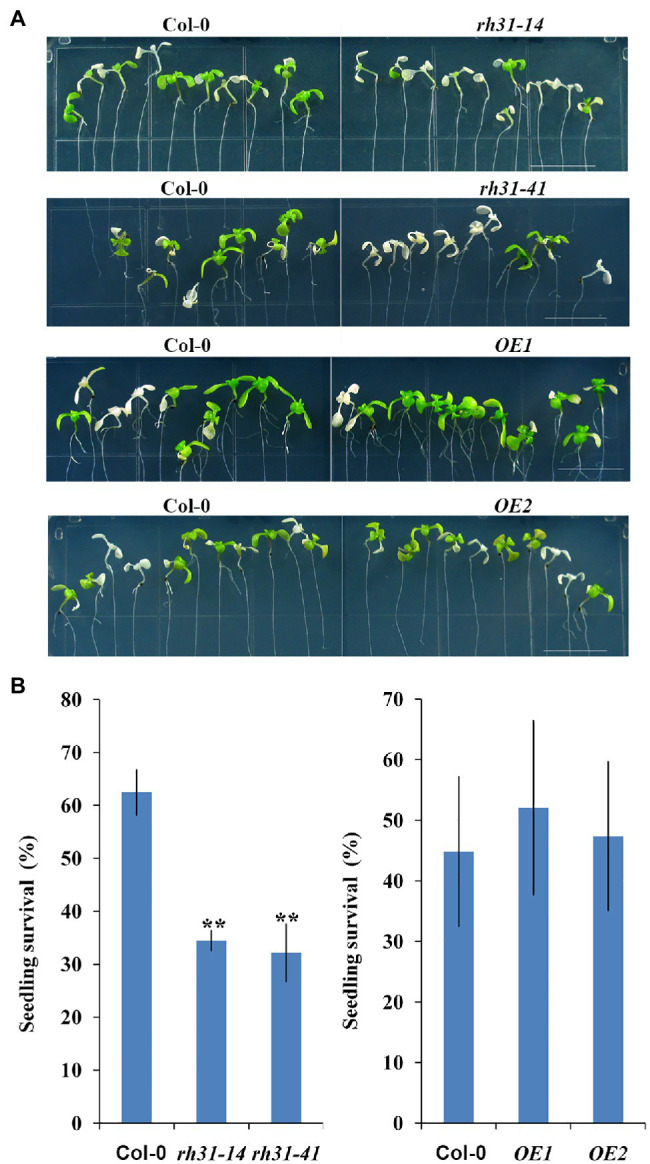
Survival Phenotype characterization of *rh31* and *RH31* overexpression lines (*OE*1, *OE*2). **(A)** Survival phenotype of Col-0, *rh31-14*, *rh31-41*, and 35S: RH31 seedlings under salt treatment. Plants were grown on 1/2 MS medium for 6 days and then transferred onto 1/2 MS medium supplemented with 200 mM NaCl for 5 days. Experiments were repeated three times with similar results. Scale bars = 1 cm. **(B)** Survival rate of seedlings shown in **(A)**. At least 30 seedlings were examined per replicate (*n* = 3 replicates). ^*^p < 0.05 and ^**^p < 0.01 (Student’s *t*-test) indicate significant differences between mutants or transgenic lines and Col-0 plants. Error bars indicate the standard error for the average of three independent experiments.

When Col-0, *rh31*, and *OE* seedlings growing on 1/2 MS medium were transferred to 1/2 MS medium containing 200 mM NaCl for 5 days, the seedling survival rate of *rh31* was significantly lower than that of Col-0 (Figure 6). The leaves of *rh31* were chlorotic and significantly bleached as compared to those of Col-0, while the leaves of *OE*s displayed slight greenish than Col-0 (Figure 6A), and *rh31* seedlings showed only 34.4% (*rh31-14*) and 32.1% (*rh31-41*) of plants surviving, compared with 62.5% of Col-0 seedlings surviving (Figure 6B). Using trypan blue staining assay, we found that a significant number of cells in *rh31* mutants was positively stained after salt treatment, indicating more cells were dead, while for *OE*s, fewer positive cells were stained, indicating less cells were dead (Supplementary Figure S3).

To confirm that the observed *rh31* mutant phenotype indeed results from *RH31* deletion, a complementation line of *RH31* (*4–2*) was generated. As shown in Supplementary Figure S4, the phenotype of the *4–2* complementation line was similar to those of Col-0. The above results suggest that *RH31* deletion results in hypersensitivity to salt, i.e., RH31 functions as a positive regulator of salt-stress tolerance in *Arabidopsis*.

### RH31 Affects Expression of Several Salt-Inducible Genes in Response to Stress

Previous work indicated that TSN1 regulates growth under stress by modulating the transcriptional level of *GA20ox3* in *Arabidopsis* (Yan et al., 2014). As its binding protein, RH31 might also be involved in this regulation pathway. We tested *GA20ox3* mRNA level in *rh31*, but unexpectedly, no significant difference was observed between Col-0 and *rh31* (Supplementary Figure S5), indicating that RH31 might function in other pathway than regulating *GA20ox3* mRNA level.

**Figure 7:**
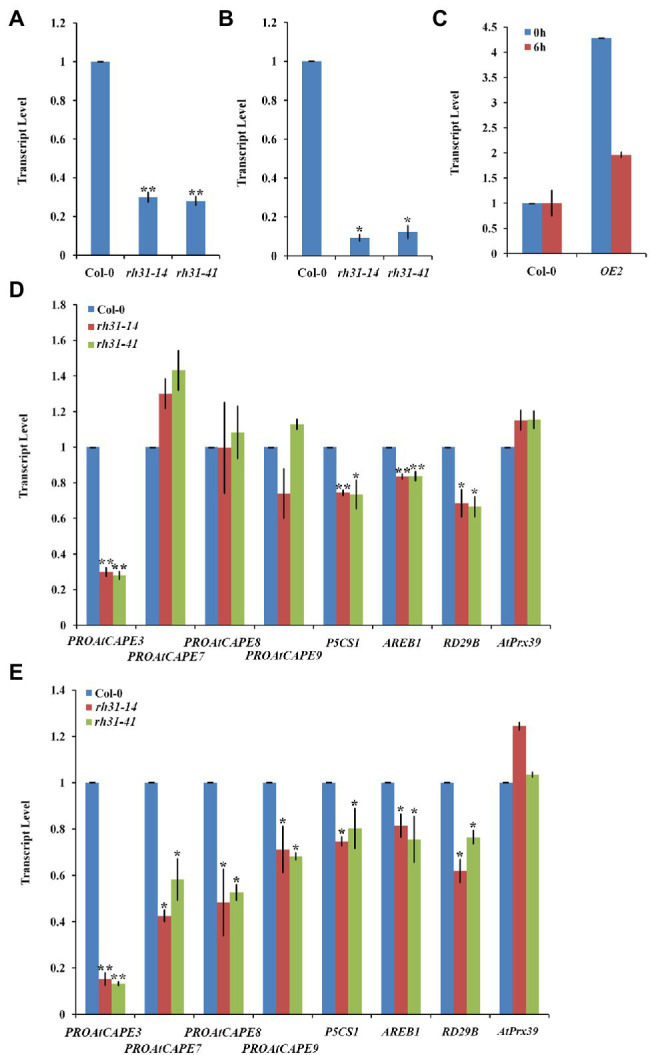
*PROAtCAPE3* and salt-inducible gene expression analysis under normal and salt-stress conditions. **(A,B)** Quantitative RT-PCR of *PROAtCAPE3* in *rh31* and Col-0 lines under normal growth condition **(A)** or after treatment with 150 mM NaCl for 24 h **(B)**. **(C)** Quantitative RT-PCR of *PROAtCAPE3* in *RH31* overexpression (*OE*2) and Col-0 lines under normal growth condition or after treatment with 150 mM NaCl for 6 h. **(D,E)** Quantitative RT-PCR of *PROAtCAPE3* and salt-inducible genes in *rh31* and Col-0 lines under normal growth condition **(D)** or after treatment with 150 mM NaCl for 3 h **(E)**. Each value indicates relative quantity, with the genes expressed in Col-0 set at 1.0. ^*^p < 0.05 and ^**^p < 0.01 (Student’s *t*-test) indicate significant differences between mutants or transgenic lines and Col-0 plants. Error bars indicate the standard error for the average of three independent experiments.

To understand how RH31 regulates salt tolerance in *Arabidopsis*, the expression levels of various salt-inducible genes, based on transcriptome profiling analysis results (Peng et al., 2014), were tested in Col-0 and *rh31* mutant seedlings. 7-day-old seedlings were treated with or without 150 mM NaCl for 24 h. We found that under normal condition, of these detected genes, *PROAtCAPE3* (At4g33720), whose translation protein PROAtCAPE3 is classified as one of the *c*ysteine-rich secretory proteins, antigen 5, and patho-genesis-related 1 proteins (CAP) superfamily (Chien et al., 2015), was decreased upon *RH31* deletion. Quantitatively, in contrast with Col-0, the transcript levels of *PROAtCAPE3* in *rh31-14* and *rh31-41* were reduced by 70 and 72%, respectively (Figure 7A). However, when seedlings were treated with 150 mM NaCl for 24 h, compared to Col-0, the *PROAtCAPE3* transcript levels in *rh31-14* and *rh31-41* were reduced by 91 and 88%, respectively (Figure 7B), which was in consistent with the phenotype of *rh31* grown under salinity (Figures 5D–F; Supplementary Figure S2). In addition, the transcript levels of *PROAtCAPE3* were increased in *RH31*-*OE*s under both normal and salt-stress conditions (Figure 7C). These results indicate that *PROAtCAPE3* mRNA levels can be significantly affected by *RH31* deletion, an effect that is exacerbated under salinity. But the expression of other salt-inducible genes we tested such as At1g49570 and At5g19890, both encoding a peroxidase, and At1g60810, encoding subunit A of the trimeric enzyme ATP Citratelyase, and *ATL31* (At5g27420) did not change significantly (Supplementary Figure S6). Nine potential PROAtCAPEs were identified as precursor candidates for AtCAPEs in *Arabidopsis* (Chien et al., 2015). We thus determined the mRNA levels of these nine *PROAtCAPEs*. Under normal condition, *PROAtCAPE7* (At2g14580), *PROAtCAPE8* (At5g26130), and *PROAtCAPE9* (At2g14610) mRNA levels were not affected by RH31 deletion (Figure 7D). However, after treatment with 150 mM NaCl, the mRNA levels of both *PROAtCAPE7* and *PROAtCAPE8* were decreased by approximately twofold, and *PROAtCAPE9* mRNA level was decreased by approximately 1.5-fold (Figure 7E). *AREB1* (At1g45249; Fujita et al., 2005) in Col-0 was highly increased by salt treatment by approximately 6-fold as compared to normal condition, which was consistent with previous findings (Chien et al., 2015). However, *rh31* mutation resulted in a slight decrease in *AREB1* as compared to Col-0 under salinity (Figure 7E).

As reported *P5CS1* (At2g39800) and *RD29B* (At5g52300), involve in osmoprotectant biosynthesis, are high-salt-inducible downstream genes (Yoshiba et al., 1999; Uno et al., 2000). In our tests, *P5CS1* and *RD29B* mRNA levels were also decreased by *RH31* deletion (Figure 7E). The above results suggest that *RH31* deletion affects the transcript levels of *PROAtCAPE3* and some other salt-inducible genes.

### PROAtCAPE3 Positively Regulates Salt Tolerance

**Figure 8:**
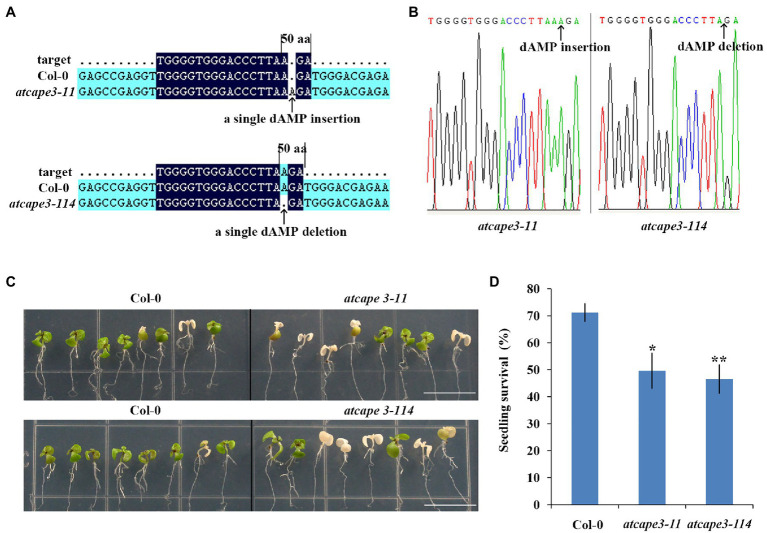
Generation of *atcape3* mutants and seedling survival rate. **(A)** Schematic of the *atcape3* mutant insertion (*atcape3-11*) site and deletion (*atcape3-114*) site. Arrows point to the insertion site and deletion site. **(B)** SANGER sequencing chromatography confirming insertion site and deletion site in *atcape3* mutants. Arrows point to the insertion site and deletion site. **(C)** Survival phenotype of Col-0 and *atcape3* seedlings under salt treatment. Plants were grown on 1/2 MS medium for 6 days and then transferred onto 1/2 MS medium supplemented with 200 mM NaCl for 5 days. Experiments were repeated three times with similar results. Scale bars = 1 cm. **(D)** Survival rate of seedlings shown in **(C)**. At least 30 seedlings were examined per replicate (*n* = 3 replicates). ^*^p < 0.05 and ^**^p < 0.01 (Student’s *t*-test) indicate significant differences between mutants and Col-0 plants. Error bars indicate the standard error for the average of three independent experiments.

The significant reduction of *PROAtCAPE3* expression in *rh31* prompted us to hypothesize that *rh31* salt sensitivity might be mediated through *PROAtCAPE3* downregulation. To test the role of PROAtCAPE3 in response to salt stress, we constructed *PROAtCAPE3* knockout mutants *atcape3-11* and *atcape3-114* (Figures 8A,B) and compared the salt tolerance of Col-0 and *atcape3* mutants under salinity (Figure 8C). In the presence of 200 mM NaCl, *atcape3* seedlings showed only 49.6% and 46.5% of plants surviving after salt exposure, compared with 71.1% of Col-0 seedlings surviving (Figure 8D). These results indicate that PROAtCAPE3 acts positively in the salt tolerance response.

## Discussion

### RH31 Is the Binding Protein of TSN1 and Localizes in SGs Under Stress Conditions

SGs are ubiquitous and assemble through protein-RNA, protein–protein, and RNA–RNA interactions. The composition of SGs differ according to stressor and cell type (Anderson and Kedersha, 2008), but some core components are conserved (Kedersha et al., 2002). SGs also contain RNA-binding proteins (Anderson and Kedersha, 2008) and RHs (Yu et al., 2011; Yasuda-Inoue et al., 2013), such as numerous ATP-dependent helicases and protein remodelers are identified in purified yeast and mammalian stress granule cores (Jain et al., 2016). Proteins involved in various aspects of mRNA metabolism and diverse cell signaling pathways are also the components of SGs (Kim et al., 2005).

As a major constituent of SGs, TSN functions in mRNA decapping during heat-stress tolerance (Gutierrez-Beltran et al., 2015). TSN is also involved in regulating transcription (Paukku et al., 2003; Yang et al., 2014), pre-mRNA splicing (Yang et al., 2007), mRNA stabilization (Paukku et al., 2008), RNA silencing (Caudy et al., 2003), and cleaving hyperedited double-stranded RNA (Scadden, 2005) in animals. Most relevant to the present study, TSN is established with an important role in stress signaling as a docking platform for stress granule proteins and is necessary for assembly and/or function of SGs (Gao et al., 2015; Gutierrez-Beltran et al., 2021).

Protein–protein interaction is important for RHs to accomplish their functions (Sugiura et al., 2007). In this study, we found a DEAD-box RH, RH31, which is a TSN1 binding protein (Figure 1). More importantly, RH31 is a component of plant SGs through fluorescence co-localization and CHX treatment experiments (Figure 3). Under stress condition, RH31 translocates from the nucleus to cytoplasm, where it localizes to SGs (Figures 2,3).

It has been reported that RHs function in a variety of RNA metabolism processes and are known as the best candidates for RNA chaperones (Richardson et al., 1998; Tanner and Linder, 2001; Carlotto et al., 2016). The DEAD-box RH AhRH47 maintains protein synthesis to enhance tolerance to salinity and mannitol-induced stresses (Mahesh and Udayakumar, 2018). DDX21 translocates from nucleus to cytoplasm, activating the innate immune response against Dengue Virus through stimulating IFN-β induction and consequently hinders Dengue Virus replication in the early phases of infection (Dong et al., 2016; Mohamed, 2021). As reported before, RNA binding protein CIP29 interacts with DEAD box RH DDX39 to enhance its helicase activity (Sugiura et al., 2007). Therefore, as a DEAD-box RH, RH31 may transfer from the nucleus to cytoplasm under salt stress, binding, and stabilizing adversity-related mRNAs, while the interacting partner-RNA binding protein TSN1 may enhance RH31 helicase activity and promote SGs formation.

### RH31 Positively Regulates Salt-Stress Tolerance, Probably by Upregulating *PROAtCAPE3*, and Some Other Salt-Inducible Genes in *Arabidopsis*

The knockout mutant *rh31* showed a salt-hypersensitive phenotype, while *RH31-OE*s conferred salt tolerance to *Arabidopsis* seedlings and the complemented transgenic line restored the wild-type phenotype (Figures 5,6; Supplementary Figures S2–S4). Thus, RH31 was identified as a positive regulator of salt tolerance in *Arabidopsis*. By qRT-PCR analysis, we determined genes that were changed significantly in *RH31-OE*, *rh31*, and Col-0 (Figure 7). Among the detected genes, the transcript level of *PROAtCAPE3* was obviously reduced in *rh31* (Figures 7A,B) while increased in *RH31*-*OE*s (Figure 7C).

As reported before, nine potential CAPs are identified in *Arabidopsis*, which are named PROAtCAPEs, and the peptide derived from PROAtCAPE is designated AtCAPE (Chen et al., 2014; Chien et al., 2015). As a member of CAP superfamily, *Arabidopsis* pathogenesis-related protein 1 (PR1, AtCAPE9) can resist *Pseudomonas syringae* pv. *tomato* strain DC3000 infection (Baek et al., 2010; Chen et al., 2014). Some PR genes are activated by abiotic stresses, suggesting that they play a role in cellular processes other than pathogen resistance (Seo et al., 2008). Additional evidence shows that *OsPR4a* overexpression in rice enhances tolerance to drought (Wang et al., 2011), and *PR1* upregulation in *Arabidopsis* confers drought tolerance (Liu et al., 2013). Overexpression of tomato PROSYSTEMIN, the precursor of the anti-herbivore systemin peptide signal (Pearce et al., 1991), shows more tolerance to salt stress (Orsini et al., 2010). Thus, plant peptides function not only in regulating innate immunity, but also in abiotic stress tolerance responses. Studies by Peng et al. (2014) indicated that *PROAtCAPE3* transcription is reduced in *ein3eil1* mutants, but increased in *EIN3ox*, and this trend is exaggerated in *ein3eil1* under salt stress. EIN3/EIL1 enhances salt tolerance by regulating myriad salt-inducible EIN3/EIL1-dependent genes. As an EIN3-induced gene, *PROAtCAPE3* may play an important role in salt stress. Indeed, our work showed that *atcape3* mutants displayed a reduced salt-tolerant phenotype (Figures 8C,D). Therefore, results of *PROAtCAPE3* downregulation can be linked with salt hypersensitivity in *rh31* mutants.

In addition to *PROAtCAPE3*, some other of the salt-inducible genes including *AREB1*, *P5CS1*, and *RD29B* were affected by RH31 deletion. The transcript level of *AREB1*, which controls the transcription of downstream ABA-dependent and salt-responsive genes and functions mainly at the vegetative stage (Nakashima and Yamaguchi-Shinozaki, 2013), is highly upregulated in Col-0 under salinity. However, *RH31* deletion led to a slight decrease in *AREB1* as compared to Col-0 (Figure 7E). As targets of AREB1, *P5CS1*, the gene for the enzyme involved in osmoprotectant biosynthesis, and the dehydration response gene, *RD29B*, were also reduced (Figure 7). These results indicate that *RH31* is necessary to maintain the transcript levels of *PROAtCAPE3* and some other salt-inducible genes.

SGs formation in mammals is involved in regulating mRNA stability and may be required for the optimal translation of stress-responsive mRNAs (Buchan and Parker, 2009; Vanderweyde et al., 2013). DEAD box RH DDX5 interacts with mettl3 to stabilize *MSR1* mRNA in macrophages and plays a positive role in macrophage lipid uptake (Zhao et al., 2018). Human DEAD-box RH DDX6 acts as an oncogene in GC cells by associating with *c-Myc* mRNA and promoting *c-Myc* expression (Taniguchi et al., 2018). Thus, mRNA stabilization during cellular stress requires specific protein–mRNA interactions (Stohr et al., 2006). Here, we showed that RH31 was a component of plant SGs (Figure 3). In addition, we found that RH31 was involved in regulating *PROAtCAPE3* mRNA levels under normal condition, an effect that was exacerbated under salinity (Figure 7). Furthermore, the *atcape3* mutant showed the same salt hypersensitivity phenotype as *rh31* mutant (Figures 6, 8C). Thus, one hypothesis is that, as a component of SGs, RH31 enhances salt tolerance by maintaining the optimal transcript levels of *PROAtCAPE3* and some other salt tolerance genes under salt stress. However, at present, we cannot rule out the possibility that RH31 increases salt tolerance through the nuclear-localized portion. The potential mechanism by which RH31 regulates salt tolerance needs to be further investigated.

## Data Availability Statement

The datasets presented in this study can be found in online repositories. The names of the repository/repositories and accession number(s) can be found in the article/Supplementary Material.

## Author Contributions

YH conceived the research, supervised the experiment. YL designed and performed the experiments, analyzed the data, and prepared the figures. SL conducted the pull down and mass spectrometry analyses. HS, JM, and MJ provided technical assistance. YL and YH wrote the manuscript. All authors contributed to the article and approved the submitted version.

## Funding

The study was supported by a grant from the National Natural Science Foundation of China (No. 31570254).

## Conflict of Interest

The authors declare that the research was conducted in the absence of any commercial or financial relationships that could be construed as a potential conflict of interest.

## Publisher’s Note

All claims expressed in this article are solely those of the authors and do not necessarily represent those of their affiliated organizations, or those of the publisher, the editors and the reviewers. Any product that may be evaluated in this article, or claim that may be made by its manufacturer, is not guaranteed or endorsed by the publisher.

## Footnotes

^1^ http://smart.embl-heidelberg.de/

^2^ http://smart.embl-heidelberg.de/
