# Supplementary material for: The TSN1 Binding Protein RH31 Is a Component of Stress Granules and Participates in Regulation of Salt-Stress Tolerance in Arabidopsis
Source: Front Plant Sci. 2021 Dec 23;12:804356. doi: 10.3389/fpls.2021.804356 (PMC8733394; doi:10.3389/fpls.2021.804356)
Supplement: Supplementary file 1 [file Data_Sheet_1.docx]

**SUPPLEMENTARY TABLE S1. Primers used in this study for genotyping, plasmid construction, RT-PCR and qRT-PCR.**

| **Primers used for genotyping** | | |
| --- | --- | --- |
| Primers | oligonucleotide | restriction site |
| RH31-crispr-LP | AACGGTCCTCCTCTGTTCCTT | MfeI |
| RH31-crispr-RP | CATTCTCCTTATCAAACCCAAA |  |
| PROAtCAPE3-crispr-LP | GCCATAACCTTTTTCCTTGTTCTCA | AflII |
| PROAtCAPE3-crispr-RP | GTGATAAAGGTTTGACCATTGTTGC |  |
| zCas9-IDF3-2 | CTGTTCGTCGAGCAGCACAAGCATT |  |
| rbcS_E9t-IDR | CATTAGAGGCCACGATTTGACACAT |  |

| **Primers used for plasmid construction** | | |
| --- | --- | --- |
| Primers | oligonucleotide | restriction site |
| RH31-OE-LP | CTGCAGTAGTGTATTGTTCTAAATGTTCTTCAACTTCCTTCC | PstI KpnI |
| RH31-OE-RP | GGTACCCTTGGCTCTAAGACCAGGAACATTTTTGA |  |
| RH31-GFP-LP | CTGCAGTAGTGTATTGTTCTAAATGTTCTTCAACTTCCTTCC | PstI KpnI |
| RH31-GFP-RP | GGTACCCTTGGCTCTAAGACCAGGAACATTTTTGA |  |
| tu1 I-1-LP | AAAGGATCCATGGCGACTGGGGCAG | BamHI EcoRI |
| tu1 I-1-RP | GCCGAATTCATGAATTGCCTTGGAGTTGC |  |
| tu1 I-2-LP | AAAGGATCCGACCAGAATTTTACCGGAAAGGTA | BamHI EcoRI |
| tu1 I-2-RP | GCCGAATTCGTTTACTACTTCCTCTCCTTCAACATAG |  |
| tu1 II-LP | AAAGGATCCGGTAGTTCTAAAGTAGAAACCAGGC | BamHI EcoRI |
| tu1 II-RP | GCCGAATTCTTGGATATCTCCGTACTCCCAG |  |
| RH31-hy-LP | AAAGAATTCATGGGAGATATTGGGGAT | BamHI EcoRI |
| RH31-hy-RP | GCCGGATCCTTACTTGGCTCTAAGACCAG |  |
| TSN1-Nluc-LP | AATTGGTACCATGGCGACTGGGGCAGCAACTG | KpnI SalI |
| TSN1-Nluc-RP | AATTGTCGACCCCGCGACCCGGTTTCCTGACTG |  |
| Cluc-RH31-LP | GGTACCTAGTGTATTGTTCTAAATGTTCTTCAACTTCCTTCC | KpnI PstI |
| Cluc-RH31-RP | CTGCAGTTACTTGGCTCTAAGACCAGGAACATTTTTG |  |

| **Primers used for RT-PCR** | |
| --- | --- |
| Primers | oligonucleotide |
| RH31-LP | CTCAAAGACCATATCGAGAACACGC |
| RH31-RP | GGGTCACATCAGGGTAATCAACTCC |
| Actin2-LP | CACTGTGCCAATCTACGAGGGT |
| Actin2-RP | GCTGGAATGTGCTGAGGGAAG |

| **Primers used for qRT-PCR** | |
| --- | --- |
| Primers | oligonucleotide |
| RH31-qLP | TGGAAGGCTCAAAGACCATATC |
| RH31-qRP | CCTTGCGAAAGCCCATATCTA |
| PROAtCAPE3-qLP | GCTGCTTATGCCCGTAACT |
| PROAtCAPE3-qRP | CCAAGCGATGTTCTCTCCATAG |
| PROAtCAPE7-qLP | CGGAACTACGCAAACCAACTA |
| PROAtCAPE7-qRP | CAAGTCACCGCCACTCTTT |
| PROAtCAPE8 -qLP | GAGCAATACGCCTGGAACTAT |
| PROAtCAPE8 -qRP | TCACCGTAAAGCCCATTTGA |
| PROAtCAPE9 -qLP | ATACACTCTGGTGGGCCTTA |
| PROAtCAPE9 -qRP | GCGTAGTTGTAGTTAGCCTTCTC |
| P5CS1-qLP | CTTGTGATACGGATATGGCAAAGCG |
| P5CS1-qRP | CCTTGGTCCACCATACAAAGTGACTCC |
| AREB1-qLP | GGATCATGGAAATGCAAAAGAAT |
| AREB1-qRP | TTGGACCTCCTTGCAGAAGATT |
| RD29B-qLP | GTGAAGATGACTATCTCGGTGG |
| RD29B-qRP | CACCACTGAGATAATCCGATCC |
| GA20ox3-qLP | AGGAGAAGATCCATTCCCAAAC |
| GA20ox3-qRP | GCCTCCGCGTATTCTTGATAA |
| AtPrx39-qLP | CACGACTCTGATAACCCTCTTTG |
| AtPrx39-qRP | GGAGCAATGAGACACTCCAATC |
| At1g49570-qLP | CGATTCTATGACCGGTCTTG |
| At1g49570-qRP | TTTGGCTGAGCGTTCTTCTC |
| At5g19890-qLP | TCTTCCTAATGCCACTCTCC |
| At5g19890-qRP | ATGGAGCTGACAGCGAGAAA |
| At5g27420-qLP | CCGTGTCGGCGGGTCAACCCGG |
| At5g27420-qRP | CTTCTGTGTCTTCACTTCTGAG |
| At5g44440-qLP | TACCCGAGGCTGCAATTCAA |
| At5g44440-qRP | CTTGTCTTCCTCTTCTCTCC |
| At1g60810-qLP | GCTCGGAAGAAGATCAGAGA |
| At1g60810-qRP | TTGCCACGCTTTCCGAACAA |
| At4g34270-qLP | GTGAAAACTGTTGGAGAGAAGCAA |
| At4g34270-qRP | TCAACTGGATACCCTTTCGCA |


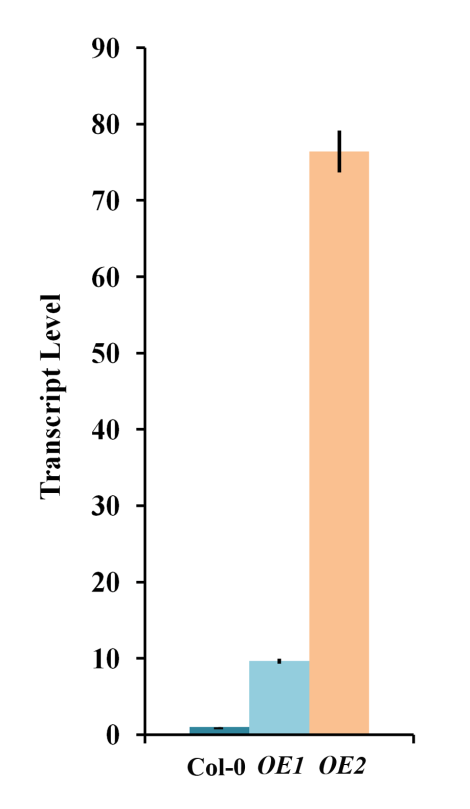


**FIGURE S1. The expression levels of *RH31* detected by quantitative RT-PCR in *RH31* overexpression lines (*OE*1, *OE*2) and Col-0 plants.** Error bars indicate the standard error for the average of three independent experiments.


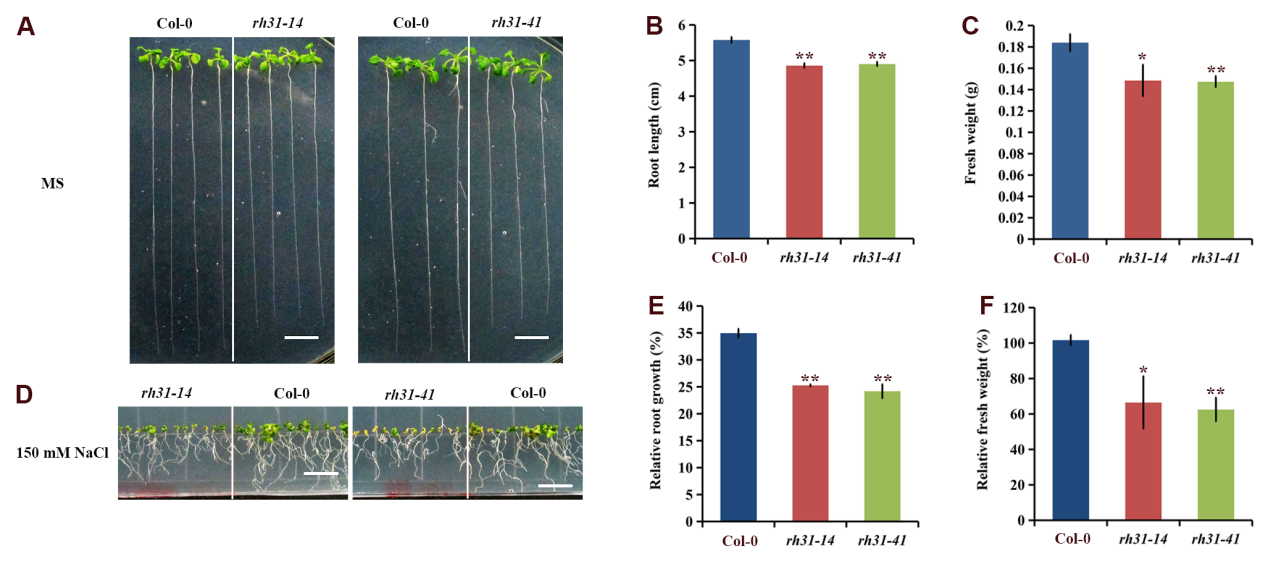


**FIGURE S2. Phenotype characterization of 20-day-old Col-0 and *rh31* seedlings.**

1. Phenotypes of 20-day-old *rh31* seedlings under normal conditions. After germinated for 24 h under normal condition, synchronized growth of Col-0 and *rh31* seedlings were transferred to MS medium for another 19 days. Experiments were repeated three times with similar results. Scale bars = 1 cm. **(B)** Root length of seedlings shown in **(A)**. The primary roots of at least 30 seedlings were measured and reported as the mean length, (n = 3 replicates). **(C)** Fresh weight of seedlings shown in **(A)**. 30 seedlings were measured per replicate, (n = 3 replicates). **(D)** Phenotypes of *rh31* seedlings treated with 150 mM NaCl for 19 days. After germinated for 24 h under normal condition, synchronized growth of Col-0 and *rh31* seedlings were transferred to MS medium with or without 150 mM NaCl for another 19 days. Experiments were repeated three times with similar results. Scale bars = 1 cm. **(E)** Relative root growth of seedlings shown in (**D**). The primary roots of at least 30 seedlings were measured and relative growth was reported as the mean length, (n = 3 replicates). **(F)** Relative fresh weight of seedlings shown in (**D**). 60 seedlings were measured under salt stress while 30 seedlings were measured under normal condition per replicate, and relative fresh weight was reported as the mean fresh weight (n = 3 replicates). **P*<0.05 and ***P*<0.01 (Student’s *t*-test) indicate significant differences between *rh31* and Col-0 plants. Error bars indicate the standard error for the average of three independent experiments.


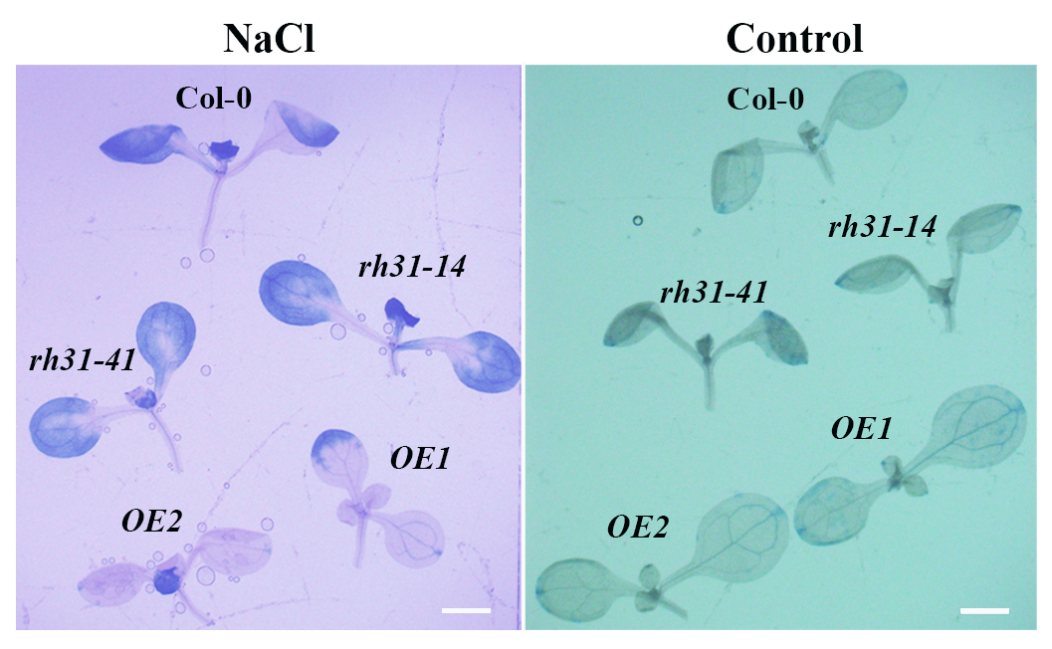


**FIGURE S3. Cell death induced by salt in cotyledons of Col-0, *rh31-14*, *rh31-41* and 35S:RH31 seedlings.** Cotyledons of 8-day-old seedlings were treated with 150 mM NaCl and stained with trypan blue. The leaves treated with ddH_2_O were used as control. Scale bars = 1 mm.


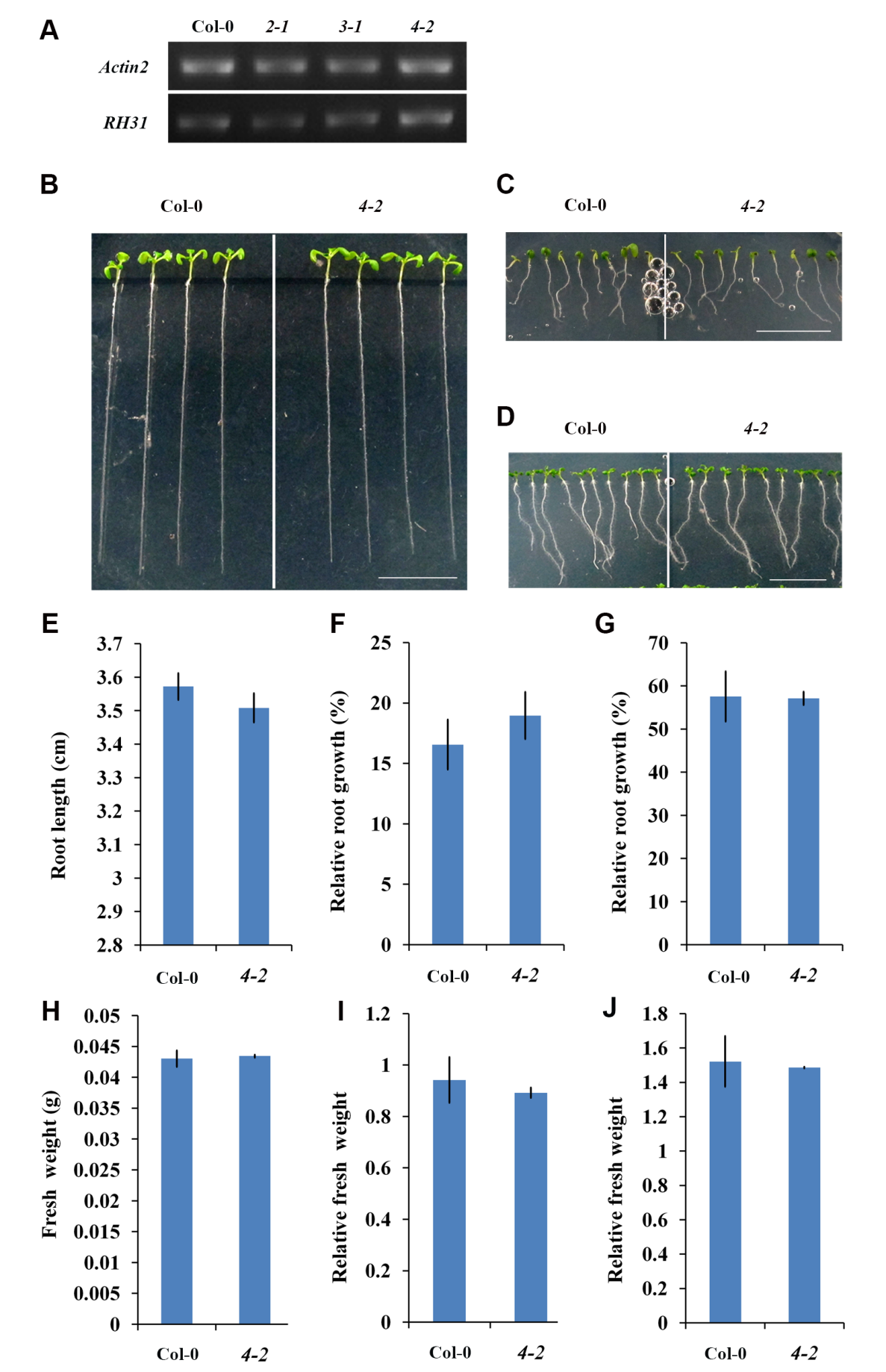


**FIGURE S4. Growth phenotype characterization of complemented transgenic line seedlings under normal and stress conditions. (A)** *RH31* expression detected by RT-PCR in *RH31* complemented transgenic lines and Col-0 plants. *4-2* was selected for further study. **(B)** Phenotypes of 7-day-old seedlings under normal condition. After germinated for 24 h under normal condition, synchronized growth of Col-0 and *rh31* seedlings were transferred to MS medium for another 10 days. Experiments were repeated three times with similar results. Scale bars = 1 cm. **(C, D)** Phenotypes of seedlings treated with 150 mM NaCl **(C)** or 250 mM mannitol **(D)** for 10 days. After germinated for 24 h under normal condition, synchronized growth of Col-0 and *rh31* seedlings were transferred to MS medium with or without 150 mM NaCl or 250 mM mannitol for another 10 days. Experiments were repeated three times with similar results. Scale bars = 1 cm. **(E)** Root length of seedlings shown in **(B)**. The primary roots of at least 30 seedlings were measured and reported as the mean length, (n = 3 replicates). **(F, G)** Relative root growth of seedlings shown in **(C)** and **(D)**. The primary roots of at least 30 11-day-old seedlings were measured and relative growth was reported as the mean length, (n = 3 replicates).**(H)** Fresh weight of seedlings shown in **(B).** 30 seedlings were measured per replicate, (n = 3 replicates). **(I, J)** Relative fresh weight of seedlings shown in **(C)** and **(D).** 30 seedlings were measured per replicate, and relative fresh weight was reported as the mean fresh weight (n = 3 replicates). **P*<0.05 and ***P*<0.01 (Student’s *t*-test) indicate significant differences between *4-2* and Col-0 plants. Error bars indicate the standard error for the average of three independent experiments.


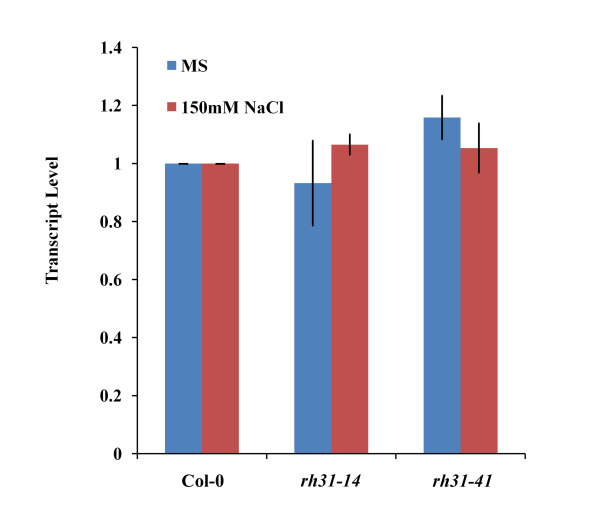


**FIGURE S5. *GA20ox3* expression analysis in Col-0 and *rh31* under normal condition or 150 mM NaCl treatment.** *RH31* deletion did not alter *GA20ox3* mRNA level. Each value indicates relative quantity, with the genes expressed in Col-0 set at 1.0. **P*<0.05 and ***P*<0.01 (Student’s *t*-test) indicate significant differences between mutants and Col-0 plants. Error bars indicate the standard error for the average of three independent experiments.


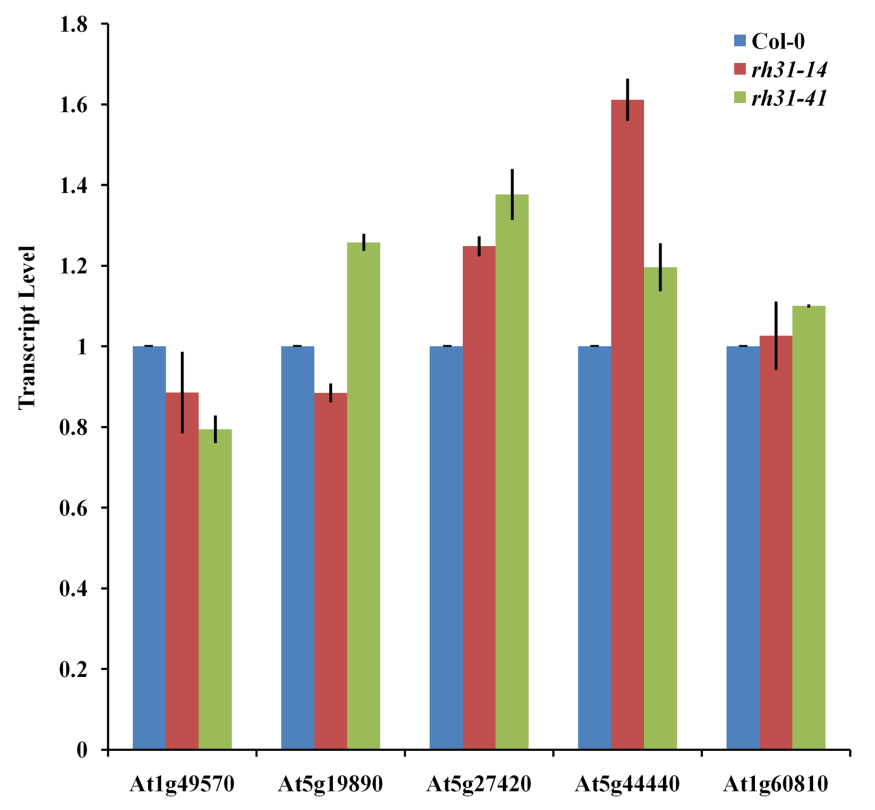


**FIGURE S6. Some salt-inducible genes analysis in Col-0 and *rh31* under normal condition.** The transcripts level of these genes in *rh31* was not affected. Each value indicates relative quantity, with the genes expressed in Col-0 set at 1.0. **P*<0.05 and ***P*<0.01 (Student’s *t*-test) indicate significant differences between mutants and Col-0 plants. Error bars indicate the standard error for the average of three independent experiments.
